# Draft Genome Sequence of *Acinetobacter baumannii* Strain MSP4-16

**DOI:** 10.1128/genomeA.00137-13

**Published:** 2013-04-04

**Authors:** Nitin Kumar Singh, Shailesh Kumar, Gajendra Pal Singh Raghava, Shanmugam Mayilraj

**Affiliations:** a Microbial Type Culture Collection and Gene Bank (MTCC) and Bioinformatics Centre; b CSIR-Institute of Microbial Technology, Chandigarh, India

## Abstract

We report the 4.0-Mb draft genome sequence of *Acinetobacter baumannii* strain MSP4-16, isolated from a mangrove soil sample from Parangipettai (11°30′N, 79°47′E), Tamil Nadu, India. The draft genome sequence of strain MSP4-16 consists of 3,944,542 bp, with a G+C content of 39%, 5,387 protein coding genes, and 69 RNAs.

## GENOME ANNOUNCEMENT

*Acinetobacter baumannii* is the most infective human pathogen of the genus *Acinetobacter*. *A. baumannii* causes hospital-acquired infections, especially in intensive care units (ICUs); hence, it is a very troublesome nosocomial pathogen. It causes wound infections, pneumonia, bacteremia, secondary meningitis, and urinary tract infections (1). *A. baumannii* can use various compounds, such as amino acids, fatty acids, and aromatic compounds, as a carbon source, which makes this organism useful in biodegradation (2). The organism in this study was isolated from a mangrove soil sample from Parangipettai (11°30′N, 79°47′E), Tamil Nadu, India, and was designated strain MSP4-16.

The genome of *A. baumannii* strain MSP4-16 was sequenced using the Illumina-HiSeq 1000 paired-end technology and produced a total of 35,456,248 paired-end reads (insert size of 350 bp) of 101 bp. We used the NGS QC Toolkit v2.3 (3) to filter the data for high-quality (HQ) vector- and adaptor-free reads for genome assembly (cutoff read length for HQ, 70%; cutoff quality score, 20). A total of 34,887,781 high-quality vector-filtered reads (~881× coverage) were used for assembly with Velvet 1.2.08 (at a hash length of 55) (4). The final assembly contains 53 contigs with a total size of 3,944,542 bp and an *N*_50_ contig length of 192.4 kb; the largest contigs assembled measure 385.4 kb.

The draft genome (53 contigs) comprising 3,944,542 nucleotides (nt) was annotated with the help of the Rapid Annotations using Subsystems Technology (RAST) (5) and RNAmmer 1.2 (6) servers. A total of 3,729 coding sequences (CDSs), 4 rRNAs, and 65 tRNAs were predicted.

RAST annotation shows that *A. baumannii* ATCC 19606 (score 526), *A. baumannii* ACICU (score 520), *A. baumannii* AB0057 (score 512), and *A. baumannii* AYE (score 508) are the closest neighbors of the strain MSP4-16. RAST annotation also shows that strain MSP4-16 contains genes coding for glycolysis and gluconeogenesis, the tricarboxylic acid (TCA) cycle, and the pentose phosphate pathway. Strain MSP4-16 also contains genes coding for urea decomposition (allophanate hydrolase, urea carboxylase, and urease accessory proteins, i.e., UreD, UreE, UreF, and UreG). Strain MSP4-16 has genes coding for the metabolism of central aromatic intermediates: 3-oxoadipate coenzyme A (CoA)-transferase subunits A and B (EC 2.8.3.6), beta-ketoadipate enol-lactone hydrolase (EC 3.1.1.24), catechol 1,2-dioxygenase (EC 1.13.11.1), 4-carboxymuconolactone decarboxylase (EC 4.1.1.44), protocatechuate 3,4-dioxygenase alpha and beta chains (EC 1.13.11.3), maleylacetoacetate isomerase (EC 5.2.1.2), and salicylate hydroxylase (EC 1.14.13.1). In the RAST annotation, we found genes coding for resistance to antibiotics and toxic compounds: spectinomycin 9-*O*-adenylyltransferase, streptomycin 3′-*O*-adenylyltransferase (EC 2.7.7.47), arsenate reductase (EC 1.20.4.1), beta-lactamase (EC 3.5.2.6), macrolide export ATP-binding/permease protein MacB (EC 3.6.3. -), arsenic resistance protein ArsH, mercuric ion reductase (EC 1.16.1.1), and chromate transport protein ChrA.

### Nucleotide sequence accession numbers.

The draft genome sequence of *A. baumannii* strain MSP4-16 has been included in the GenBank Whole-Genome Shotgun (WGS) database under the accession no. AODW00000000. The version described in this paper is the first version, accession no. AODW01000000. Genome assembly and annotation data can be downloaded from our genomics web portal at http://crdd.osdd.net/raghava/genomesrs/.
